# Editorial: Special Issue “Massive MIMO and mm-Wave Communications”

**DOI:** 10.3390/s22020519

**Published:** 2022-01-11

**Authors:** Gianmarco Romano

**Affiliations:** Department of Engineering, University of Campania “L. Vanvitelli”, 81031 Aversa, CE, Italy; gianmarco.romano@unicampania.it

Massive multiple-input multiple-output (mMIMO) communication systems and the use of millimeter-wave (mm-Wave) bands represent key technologies that are expected to meet the growing demand of data traffic and the explosion of the number of devices that need to communicate over 5G/6G wireless networks [1,2]. In mMIMO, the number of antennas is much larger than in traditional MIMO systems with the purpose of obtaining a high spectral efficiency. The use of mm-Wave bands, thanks to the short wavelength, may help to have compact arrays that can accommodate such a large number of antennas and allow overcoming the scarcity of the available spectrum, though the signal propagation is affected by severe path loss, sensitivity to blockage, directivity, and narrow beamwidth [3]. In addition, at mm-Wave bands, the size of the cells turns out to be much smaller than that required by traditional sub-6 GHz wireless networks such as 4G networks, and small-cell heterogeneous network (HetNet) architectures are required [4].

This Special Issue presents contributions to various aspects of the design and implementation of mMIMO systems that use mm-Wave bands. Topics such as precoding techniques, channel estimation, antenna design, network coverage, and health and safety issues are addressed, and the most advanced solutions are proposed. This Special Issue is a collection of eleven papers that are briefly explained in the following.

In [5], Ahamed and Faruque address the issue of network coverage planning when mm-Wave bands are used. At such high-frequency bands, the cell radius is limited to 100 m, which is much smaller than that required by current cells, e.g. several kilometers are required in 4G networks. Therefore, the huge number of small cells poses a major challenge to network layout planning, the selection of cell site parameters, and the propagation model to ensure uniform network coverage. The authors propose an updated cell architecture with six sectors and an advanced antenna system that provides better coverage.

Ansarudin et al. in [6] consider the problem of antenna design when the base station antennas become very small in size (about 30 cm) when massive MIMO systems at the millimeter waveband are employed. They focus on aperture antennas such as a dielectric lens as a viable alternative to the traditional array antenna system with regard to the capability of performing wide angular scanning for multi-beam applications. They propose a new lens-shaping method for a thin and small lens curvature, and the result is a shaped lens developed in MATLAB that presents a multi-beam radiation pattern with small beam shape distortion for a wide-angle beam scanning operation of up to $30^{\circ }$ from the center beam.

In [7], Celaya-Echarri et al. present the results of a study on radio-frequency electromagnetic field (RF-EMF) exposure assessment from an empirical and modeling approach for a large, complex indoor setting with a high node density and traffic. They consider the coexistence of two heterogeneous networks operating at mm-Wave bands and present a novel software tool to simulate different scenarios in realistic operating conditions by considering high-node user density, beamforming, radio propagation at mm-Wave bands, the body shielding effect, the dispersive material properties of obstacles, and scatterers. The results were validated with an empirical campaign of measurements that showed good agreement with the simulated results.

In [8], Hou et al. consider the problem of channel estimation in the uplink of massive MIMO systems that employ mm-Wave signals and hybrid beamforming architectures. The estimation relies on pilots whose number increases linearly with the number of users and antennas. Hence, in massive MIMO systems, it is of interest to reduce the pilot overhead, and the authors study a solution based on a compressive-sensing-based sparse channel estimator. They propose the application of a sparse Bayesian learning algorithm that uses approximate message passing with a unitary transform, which leads to an estimator with greater accuracy and reduced pilot overhead.

In [9], Kim et al. address the issue of self-interference cancellation (SI) in full-duplex massive MIMO systems. Self-interference is due to the transmitted signal that becomes a source of unwanted interference to the transmitter, and its cancellation performances require accurate estimation of the SI channel by means of training sequences. Since the size of the SI channel matrix increases with the number of antennas, partial training strategies are studied as a viable alternative to the full training strategy, which requires a large training overhead in massive MIMO systems. The authors present an efficient partial SI-channel-training framework under limited overhead and found an optimal strategy in terms of the parameters of antenna selection and pilot allocations by formulating an optimization problem to minimize the expected residual SI power. Through numerical results, it is shown that the proposed scheme can improve the sum-rate performances compared to other simple partial training schemes with a limited training overhead.

In [10], Lee et al. consider multi-user MIMO hybrid beamforming systems operating at mm-Wave bands and focus on the problem of reducing the large overhead on the feedback that reports the channel state information at the transmitter. The authors propose a solution that exploits the sparsity of the mm-Wave MIMO channel, uses nonlinear quantization, and leads to a feedback scheme that provides more accurate recovery with the same size of feedback as that of the channel recovery scheme with a linear codebook. Furthermore, the proposed feedback scheme allows a transmitter to adopt joint optimization for the hybrid beamformer.

Li et al. in [11] consider the design of hybrid precoding in mm-Wave MU-MIMO systems where an analog beamformer is used in conjunction with a digital baseband precoder that eliminates the inter-chain interference. They propose a novel hybrid precoding structure that employs a domestic switch network leading to a more compact RF structure and consumes less energy than that required by the sub-connected hybrid precoder. They also propose a design method based on the cross-entropy method in which candidate precoding matrices are randomly generated according to a probability distribution that is updated at each iteration until convergence is reached. The resulting precoder is sufficiently close to optimal. The authors also develop a Dinkelbach-method-based algorithm to optimize the energy efficiency by selecting the number of active antennas.

In [12], Mao et al. propose a novel non-stationary millimeter-wave-geometry-based stochastic model for the unmanned aerial vehicle (UAV) to vehicle communication channel (U2V). They develop the model considering that, when employing mm-Wave systems, features such as the 3D arbitrary trajectory of both terminals, the rotation of the 3D antenna array, and the 3D beam-forming of the UAV need to be taken into account. They present a hybrid computation method of channel parameters, a geometry-based part and a data-based part, to guarantee both precision and efficiency. To validate the model, the case of an urban U2V mm-Wave communication scenario is taken as an example.

Ryu and Kim in [13] consider the problem of the optimization of energy-efficient routing and throughput maximization in small-cell heterogeneous networks (HetNets) with a mm-Wave backhaul mesh. They propose a deep-reinforcement-learning algorithm for a dual-objective problem and implement a proximal-policy (PPO)-based multi-objective algorithm using the actor–critic model that interoperates with an optimistic linear support framework to find the Pareto front of the two objectives. The numerical results show that such a solution converges successfully with increasing rewards as training is iterated.

In [14], Soares et al. present a novel complex-valued radial basis function (RBF) neural network architecture for channel estimation and symbol detection in massive MIMO-OFDM communication systems. They propose a MIMO phase transmittance RBF neural network (MIMO-PTRBF) to implement massive MIMO schemes as an alternative to the classic MIMO-OSTBC systems under maximum likelihood detection and show that it is more computationally efficient with respect to the optimal maximum likelihood decoding.

In [15], Tran and Voznak propose a new switchable coupled relay model for massive MIMO non-orthogonal multiple access (MIMO-NOMA) networks. They consider a network, with a base station and multiple devices, that employs coupled relays to extend its coverage. The base station, coupled relays, and devices are all equipped with a large number of antennas (mMIMO) and use the NOMA technique to improve the capacity of the system. For such a network, it is of interest to optimize the system performances in terms of outage probability and system throughput by properly selecting one relay to forward signals to multiple devices. The authors derive theoretical analytical results in a closed-form expression to show the effectiveness of the proposed system in terms of higher throughput and lower energy consumption and provide Monte Carlo simulations to confirm their theoretical findings.

Finally, I would like to thank all the authors for their excellent contributions and the reviewers for their fruitful comments and feedback. Furthermore, I wish to express my appreciation to the excellent support by the Editorial Board and Editorial Office of the MDPI Sensors journal.

## Data Availability

Not applicable.

## References

[1] Yang S., Hanzo L. (2015). Fifty Years of MIMO Detection: The Road to Large-Scale MIMOs. IEEE Commun. Surv. Tutor..

[2] Björnson E., Sanguinetti L., Wymeersch H., Hoydis J., Marzetta T.L. (2019). Massive MIMO is a reality—What is next?: Five promising research directions for antenna arrays. Digit. Signal Process..

[3] Uwaechia A.N., Mahyuddin N.M. (2020). A Comprehensive Survey on Millimeter Wave Communications for Fifth-Generation Wireless Networks: Feasibility and Challenges. IEEE Access.

[4] Busari S.A., Huq K.M.S., Mumtaz S., Dai L., Rodriguez J. (2018). Millimeter-Wave Massive MIMO Communication for Future Wireless Systems: A Survey. IEEE Commun. Surv. Tutor..

[5] Ahamed M.M., Faruque S. (2021). 5G Network Coverage Planning and Analysis of the Deployment Challenges. Sensors.

[6] Ansarudin F., Abd Rahman T., Yamada Y., Rahman N.H.A., Kamardin K. (2020). Multi Beam Dielectric Lens Antenna for 5G Base Station. Sensors.

[7] Celaya-Echarri M., Azpilicueta L., Rodríguez-Corbo F.A., Lopez-Iturri P., Ramos V., Alibakhshikenari M., Shubair R.M., Falcone F. (2021). Towards Environmental RF-EMF Assessment of mm-Wave High-Node Density Complex Heterogeneous Environments. Sensors.

[8] Hou S., Wang Y., Li C. (2021). Uplink Sparse Channel Estimation for Hybrid Millimeter Wave Massive MIMO Systems by UTAMP-SBL. Sensors.

[9] Kim T., Min K., Park S. (2021). Self-Interference Channel Training for Full-Duplex Massive MIMO Systems. Sensors.

[10] Lee W.S., Song H.K. (2021). Efficient Channel Feedback Scheme for Multi-User MIMO Hybrid Beamforming Systems. Sensors.

[11] Li X., Huang Y., Heng W., Wu J. (2021). Machine Learning-Inspired Hybrid Precoding for mm-Wave MU-MIMO Systems with Domestic Switch Network. Sensors.

[12] Mao K., Zhu Q., Song M., Hua B., Zhong W., Ye X. (2020). A Geometry-Based Beamforming Channel Model for UAV mm-Wave Communications. Sensors.

[13] Ryu K., Kim W. (2021). Multi-Objective Optimization of Energy Saving and Throughput in Heterogeneous Networks Using Deep Reinforcement Learning. Sensors.

[14] Soares J.A., Mayer K.S., de Castro F.C.C., Arantes D.S. (2021). Complex-Valued Phase Transmittance RBF Neural Networks for Massive MIMO-OFDM Receivers. Sensors.

[15] Tran T.N., Voznak M. (2021). Switchable Coupled Relays Aid Massive Non-Orthogonal Multiple Access Networks with Transmit Antenna Selection and Energy Harvesting. Sensors.

